# The Critical Role of N- and C-Terminal Contact in Protein Stability and Folding of a Family 10 Xylanase under Extreme Conditions

**DOI:** 10.1371/journal.pone.0011347

**Published:** 2010-06-28

**Authors:** Amit Bhardwaj, Sadhu Leelavathi, Sudeshna Mazumdar-Leighton, Amit Ghosh, Suryanarayanarao Ramakumar, Vanga S. Reddy

**Affiliations:** 1 International Centre for Genetic Engineering and Biotechnology, New Delhi, India; 2 Department of Botany, University of Delhi, Delhi, India; 3 National Institute of Cholera and Enteric Diseases, Kolkata, India; 4 Department of Physics, Indian Institute of Science, Bangalore, India; 5 Bioinformatics Centre, Indian Institute of Science, Bangalore, India; Griffith University, Australia

## Abstract

**Background:**

Stabilization strategies adopted by proteins under extreme conditions are very complex and involve various kinds of interactions. Recent studies have shown that a large proportion of proteins have their N- and C-terminal elements in close contact and suggested they play a role in protein folding and stability. However, the biological significance of this contact remains elusive.

**Methodology:**

In the present study, we investigate the role of N- and C-terminal residue interaction using a family 10 xylanase (BSX) with a TIM-barrel structure that shows stability under high temperature, alkali pH, and protease and SDS treatment. Based on crystal structure, an aromatic cluster was identified that involves Phe4, Trp6 and Tyr343 holding the N- and C-terminus together; this is a unique and important feature of this protein that might be crucial for folding and stability under poly-extreme conditions.

**Conclusion:**

A series of mutants was created to disrupt this aromatic cluster formation and study the loss of stability and function under given conditions. While the deletions of Phe4 resulted in loss of stability, removal of Trp6 and Tyr343 affected *in vivo* folding and activity. Alanine substitution with Phe4, Trp6 and Tyr343 drastically decreased stability under all parameters studied. Importantly, substitution of Phe4 with Trp increased stability in SDS treatment. Mass spectrometry results of limited proteolysis further demonstrated that the Arg344 residue is highly susceptible to trypsin digestion in sensitive mutants such as ΔF4, W6A and Y343A, suggesting again that disruption of the Phe4-Trp6-Tyr343 (F-W-Y) cluster destabilizes the N- and C-terminal interaction. Our results underscore the importance of N- and C-terminal contact through aromatic interactions in protein folding and stability under extreme conditions, and these results may be useful to improve the stability of other proteins under suboptimal conditions.

## Introduction

Thermal stability of proteins is one of the most extensively documented properties in protein biochemistry, and yet we still do not have a complete understanding of the stabilization strategies adopted by proteins. Stabilization becomes even more difficult under poly-extreme conditions, such as extreme pH, protease treatment and SDS treatment. Each class of proteins appears to have evolved its own mechanism for establishing protein stability under extreme conditions rather than converging on a single universal mechanism. It is therefore necessary to identify the determinants of protein stability for each class of proteins. The TIM-barrel structure (β/α)_8_ provides an excellent model to address the function and stability of an enzyme. It is the most common protein fold and is present in approximately 10% of all known enzyme structures. Several investigations have been carried out to better understand the principles underlying the folding and stability of the TIM-barrel fold [1], [ 2], [ 3]. The major interactions reported to be responsible for these properties include ionic bonding, hydrogen bonding and hydrophobic interactions. Aromatic interactions have also been reported to enhance the stability of proteins, mainly via dimer or higher order polymer formation [4]. Nevertheless, a recent *in silico* analysis based on protein structures available in the protein structure database has pointed out the correlation between the presence of large numbers of aromatic clusters and the thermal stability of proteins [5]. However, until recently, no experimental evidence was available concerning the significant role, if any, played by any specific aromatic interaction(s) between the N- and C-terminus in protein folding and stability under poly-extreme conditions.

BSX is an extra-cellular endo-xylanase that belongs to the glycosyl hydrolase family 10 (GH10). It is derived from an alkalophilic *Bacillus* sp. NG-27 [6]. BSX is optimally active at 343K and pH 8.4. The crystal structures of native and xylosaccharide substrate-bound BSX have been determined and refined at 2.2Å [7], [ 8]. BSX folds as a (β/α)_8_ barrel, and structural analysis has revealed the presence of a higher percentage of surface-exposed acidic residues that contribute to the alkaline stability of BSX [8]. Until recently, BSX was the only GH10 family xylanase reported to show high structural and functional stability at high temperature (optimum activity at 343K), high alkaline pH (remains active up to pH 10), and high salt concentration (retains 80% of the enzyme activity in the presence of 2 M NaCl), as well as in the presence of SDS (retains 90% activity in the presence of 2% SDS) and proteinase K treatment [8], [ 9]. The BSX gene (only the mature part, excluding 51 amino acid signal peptides) was cloned and expressed in *E. coli* for its recombinant expression and thus named R-BSX [9]. Both BSX and R-BSX were found to be identical in all bio-chemical and bio-physical aspects and hence R-BSX was used as a control [9]. Sequence homology studies have shown that the N-terminus is not highly conserved among the family of GH10 enzymes, and perhaps this could explain the basis for the differences in stability of these molecules under extreme conditions. In our quest to identify structural determinants for BSX stability under extreme conditions, we carried out a bioinformatics analysis on the BSX crystal structure data using Scide [10], [ 11] and the Protein Interaction Calculator [12]. In addition, stabilizing cation-pi interactions were also identified using the CaPTURE program [13]. Interaction between the very N- and C- terminal aromatic residues of BSX which includes Phe4 – Trp6 – Tyr343 (F-W-Y) was found to be an interesting and unique feature of this molecule compared to molecules that are thermolabile in nature. A recent study by Krishna and Englander showed that half of the proteins in the database have their N- and C-terminal elements in contact [14], and it emphasized the significance of the protein folding, native state stability and turnover of the molecules.

It is possible that the long range N- and C-terminal aromatic interactions (F-W-Y) found in BSX may be responsible for keeping the termini in close proximity, and this could be the basis for its stability under poly-extreme conditions. To test this hypothesis, a series of deletion/substitution mutants were created to disrupt the Phe4 – Trp6 – Tyr343 aromatic cluster, and each mutant protein was analyzed for its folding, stability and function at high temperature as well as in the presence of SDS and proteinase K. We also carried out limited proteolysis of BSX and all its mutant proteins with trypsin under native conditions, and the peptide mass fingerprinting was obtained using mass spectrometry. Stability correlated with structural changes due to the deletion/substitution of the respective residue [15]. For a better comparison, we also mutated non-aromatic residues at the N-terminus (Gln2 and Pro3) and studied stability of mutant proteins under similar conditions. Our study provides the biochemical and molecular evidence that the F-W-Y aromatic cluster plays a critical role in maintaining the structural integrity of BSX under poly extreme conditions.

## Materials and Methods

### Generation of site – directed mutants of R-BSX

Mutants of R-BSX (Table 1) were generated using the Quikchange® site – directed mutagenesis kit (Stratagene, La Jolla, CA), according to the manufacturer's instructions. pET14bxyla5-14 (R-BSX) was used as a template [9]. All mutants were verified by DNA sequencing.

**Table 1:** Effect of various mutations on xylanase activity and stability under various conditions.

Name	Amino acid/Replaced with	N- terminal amino-acidSequence (1^st^–6^th^ residue)	C- terminal amino-acidSequence(342^nd^–347^th^ residue)	Effect on enzyme activity	T_m_(K)	Effect on SDS stability	Effect on Proteinase K stability
BSX (Native, Extracellular)	-	VQPFAW	NYRVKP	-	∼343	Resistant	Resistant
R-BSX (Recombinant)	-	MVQPFAW	NYRVKP	-	∼343	Resistant	Resistant
ΔQ2	Gln(2)/-	MV*PFAW	NYRVKP	No change	∼343	Resistant	Resistant
ΔP3	Pro (3)/-	MVQ*FAW	NYRVKP	No change	∼341	Decreased	Decreased
ΔF4	Phe(4)/-	MVQP*AW	NYRVKP	Decreased	∼337	Decreased	Susceptible
ΔW6	Trp (6)/-	MVQPFA*	NYRVKP	No activity	N.A	Decreased	Susceptible
F4A	Phe(4)/Ala	MVQP**A**AW	NYRVKP	No change	∼339	Decreased	Resistant
F4W	Phe(4)/Trp	MVQP**W**AW	NYRVKP	No change	∼343	Increased	Resistant
W6A	Trp(6)/Ala	MVQPFA**A**	NYRVKP	No change	∼333	Decreased	Susceptible
Y343A	Tyr(343)/Ala	MVQPFAW	N**A**RVKP	No change	∼333	Decreased	Susceptible
ΔY343	Tyr (343)/-	MVQPFAW	N*RVKP	No activity	N.A	Decreased	Susceptible

Residues are numbered according to BSX (native) sequence. In column 3 and 4, Deletion and substitution of a respective residue is shown by an “asterisk” and “bold letter” respectively.

### Protein expression and purification

All mutants were expressed in BL21 (DE3) cells and then purified from the soluble fraction of the cell lysate as described previously. The biochemical properties of native BSX (from *Bacillus sp.* NG-27) and recombinant BSX (R-BSX, expressed in *E.coli*), which contains an extra Met as a start codon, were identical [9]. Therefore, R-BSX was used as a control in this study. Protein concentration was determined using the Bradford method [16]. The amino acid sequence of R-BSX and its mutants were numbered according to the BSX (native) sequence.

### Circular dichroism (CD)

CD measurements of mutant proteins were carried out with a JASCO spectropolarimeter equipped with a peltier cell holder and a PTC-348 temperature controller, as described previously for R-BSX [9]. The solvent spectrum was subtracted from the sample spectrum. The thermal denaturation curve was monitored by the decrease of CD signal at 222 nm using a heating rate of 333K/hr.

### Enzyme activity assay

Xylanase activity of R-BSX and its derivatives was determined using 1.5% oat spelt xylan (Sigma-Aldrich,USA) in 50 mM Tris-HCl (pH 8.5) as a substrate [6]. Unless otherwise stated, the assay was carried out at 338K for R-BSX and its mutants. For thermal inactivation studies, enzymes were heated for 15 min at various temperatures and assayed for residual activity at their optimum temperature, as described previously [9].

### Thermal unfolding using native PAGE

Native-Polyacrylamide gel electrophoresis (PAGE) was performed according to the method of Davis and Ornstein [17]. Protein samples were incubated for 15 min at various temperatures and then immediately placed at 277K to avoid any refolding. All samples were mixed with 4× loading dye and loaded onto a 12% native PAGE gel, as described elsewhere [9].

### SDS stability

R-BSX and mutants were incubated in various concentrations of SDS at room temperature for 12 hrs and residual activity was measured. Xylanase activity in the absence of SDS was taken as 100% activity. SDS-PAGE analysis for SDS tolerance was performed as described elsewhere [9].

### Proteinase K assay

The proteolytic resistance of R-BSX and its mutants was studied using proteolysis by proteinase K. The protein samples (1 µg) were incubated in the presence of proteinase K at 301K for 24 hr, denatured by boiling in 4× SDS-PAGE loading dye (containing SDS and 2-mercaptoethanol), and subjected to SDS-PAGE. Gels were stained using Coomassie blue.

### Pulse proteolysis of proteins in urea

Pulse proteolysis experiments were performed as described previously [18]. Briefly, R-BSX and its mutants were incubated with various concentrations of urea, at least overnight, before proteolysis. Proteolysis was initiated by adding proteinase K in 20 mM Tris-Cl, pH 8.5, to a final concentration of 0.15 mg/ml. After 1 min of incubation, the reaction was stopped by adding 2 µl of 10× complete protease inhibitor tablet solution (Roche) and the reaction mixture was immediately boiled for 5 min after adding the 4× protein sample buffer. Protein samples were loaded onto 12% (w/v) SDS-PAGE gels and run according to the Laemmli buffer system, stained with Coomassie blue dye and scanned with an image scanner (GE Biosciences). Band intensities were quantified using AlphaView™ (Alpha Innotech). C_m_, *m*-values and ΔG_unf_^o^ were calculated as described previously [18].

### *In silico* analysis

A **s**tructural model of BFX was generated by SWISS MODEL [19], a fully automated protein structure homology-modeling server, using the BSX crystal structure (PDB ID-2FGL) as a template. Structural models of various R-BSX mutants were generated using the Swiss-pdb viewer [20]. Aromatic interactions and cation-pi interactions were predicted using the Protein Interaction Calculator (PIC) [12] and CaPTURE programs [13], respectively, for BSX (PDB id – 2FGL), BHX (*Bacillus halodurans* xylanase, PDB id – 2UWF) and BFX (*Bacillus firmus* xylanase). GETAREA [21] analysis was performed to determine the solvent accessibility area of the residues.

### Limited proteolysis of BSX and its mutants using trypsin under native conditions

Limited proteolysis of BSX and its mutants using trypsin was carried out in a buffer of 10 mM Tris-Cl, pH 8.5 at 310K. The enzyme to protein ratio was adjusted to 1∶20 with 5 µg of protein present initially. The total reaction mixture volume was adjusted to 30 µl. Reactions were stopped using 0.1% TFA after an interval of 5 min, 15 min and 30 min. For MALDI analysis, the reaction mixture was vacuum dried using a speed-vac concentrator (Thermo Scientific) and resuspended in 5 µl of 0.1% TFA. 1 µl was applied to the ground steel target plate (Bruker Daltonik GmbH) with 1 µl of α-cyano-4-hydroxycinnamic acid (Bruker Daltonik GmbH) and allowed to dry (dried droplet).

### MALDI analysis

All MALDI-MS experiments were carried out using a Bruker Ultraflex III™ TOF/TOF mass spectrometer (Bruker Daltonik GmbH). The spectra were recorded in the positive mode, using the reflector mass analyzer. Calibration was initially performed by external calibration using the Bruker Peptide Standards. Data analysis was carried out using the Flex analysis and Biotools programs (Bruker Daltonik GmbH).

## Results

### Sequence conservation vs. aromatic cluster formation

**Figure 1:**
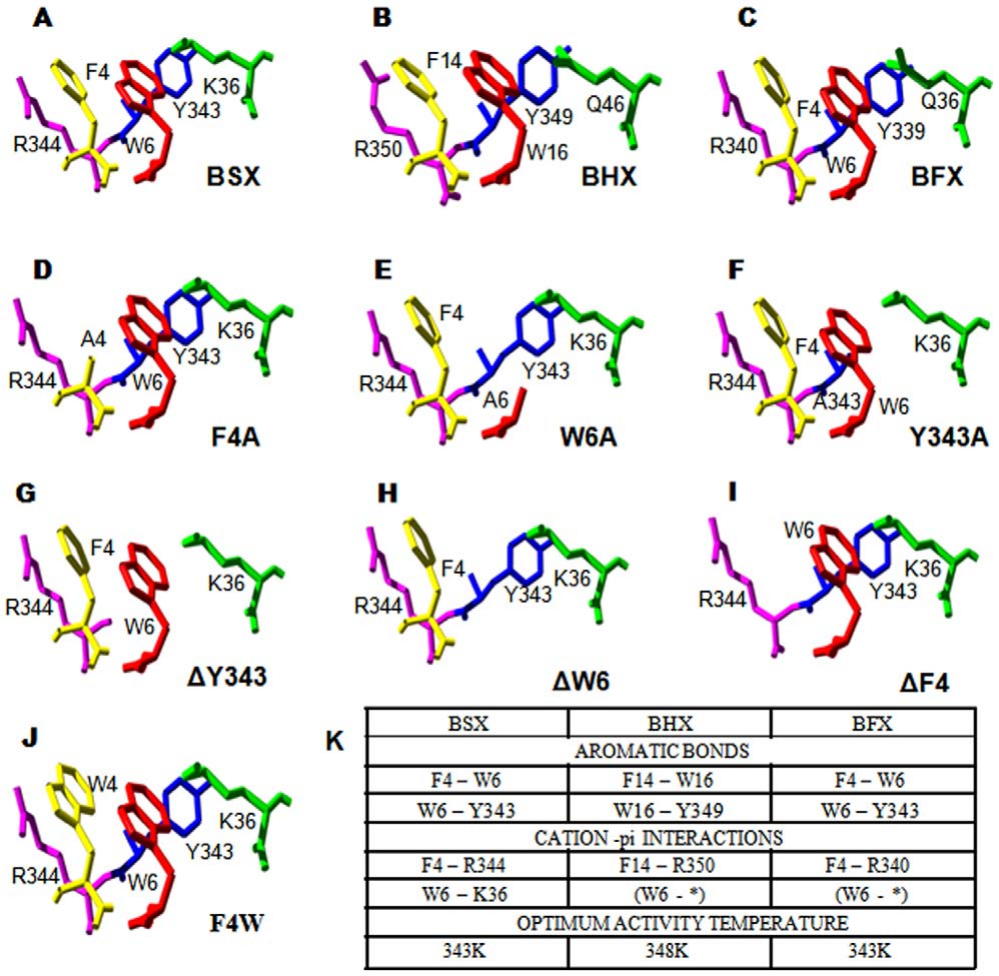
N- and C- terminus contact via aromatic interactions. The presence of conserved F-W-Y aromatic clusters in thermostable BSX (A), BHX (B), and BFX (C) are shown. Lys36 of BSX was replaced with Gln46 in BHX and Gln36 in BFX. The SWISS-pdb generated models of F4A (D), W6A (E), Y343A (F), ΔY343 (G), ΔW6 (H), ΔF4 (I) and F4W (J) are also shown. The introduction of alanine clearly disrupts the F-W-Y aromatic cluster (D, E & F) whereas replacement of Phe4 with Trp keeps the aromatic cluster intact (J). The predicted aromatic interactions, cation-pi interactions, and optimal activity temperatures for BSX, BHX and BFX are presented in tabular form (K).

Multiple sequence alignment of BSX with other GH10 xylanases revealed that the aromatic cluster of interest (F-W-Y) is not conserved in the GH10 xylanases from *Bacillus* N137 [22] and *B. alcalophilus* (Acc. no.–AAQ99279), which are reported as thermo labile in nature. However, this aromatic cluster was fully conserved in *B. halodurans* (BHX) [23] and *B. firmus* xylanases (BFX) [24], which are thermostable, like BSX. (Figure S1). Among the GH10 xylanases, BSX shows the highest identity (77%, 77%) and similarity (88%, 87%) scores with BHX and BFX, respectively. In particular, the N and C terminal sequences are highly conserved among BSX, BHX, and BFX. Structural alignment showed that the N-terminal aromatic cluster of BSX (Figure 1A) formed by Phe4, Trp6, and Tyr344 is very well conserved in BHX and BFX (Figure 1B & C). This result complements the reported optimal temperatures, which are 313K for xylanase from *Bacillus* N137, 343K for BHX, and 348K for BFX. However, the reported optimal temperature of xylanase from *B. alcalophilus* is not available (Acc. no.–AAQ99279).

CaPTURE analysis showed that Phe4 and Trp6 of BSX were also involved in cation-pi interaction with Arg344 and Lys36, respectively (Figure 1K). The aromatic interaction between Trp6 and Tyr343 and the cation-pi interaction between Phe4 and Arg344 also indicate a probable role of these N and C-terminal residues in protein folding and stability. Similar analyses for cation-pi interactions were also performed for BHX and BFX (Figure 1K). The results showed that the Phe4 – Arg340 cation-pi pair, similar to Phe4 – Arg344 of BSX, was well conserved in BHX and BFX. The cation-pi pair, similar to Trp6 - Lys36 of BSX, was not observed in BHX and BFX because Lys36 of BSX is replaced by Gln, which is a topologically equivalent residue in BHX and BFX. Thus, Trp6 of BHX and BFX was only involved in aromatic interaction with Tyr339, which is equivalent to Tyr343 of BSX. Structural models of mutants generated using the SwissPdb Viewer using BSX (PDB id-2FGL) as a parent molecule are shown in Figures 1D–J. Deletion mutants and substitution of aromatic residues (Phe4, Trp6, and Tyr343) with Ala disrupted the aromatic cluster, which in turn affected the N- to C-terminus contacts (Figure 1D–I), whereas substitution of Phe4 with Trp kept the aromatic cluster intact (Figure 1J).

### Thermal unfolding of BSX and its variants

**Figure 2:**
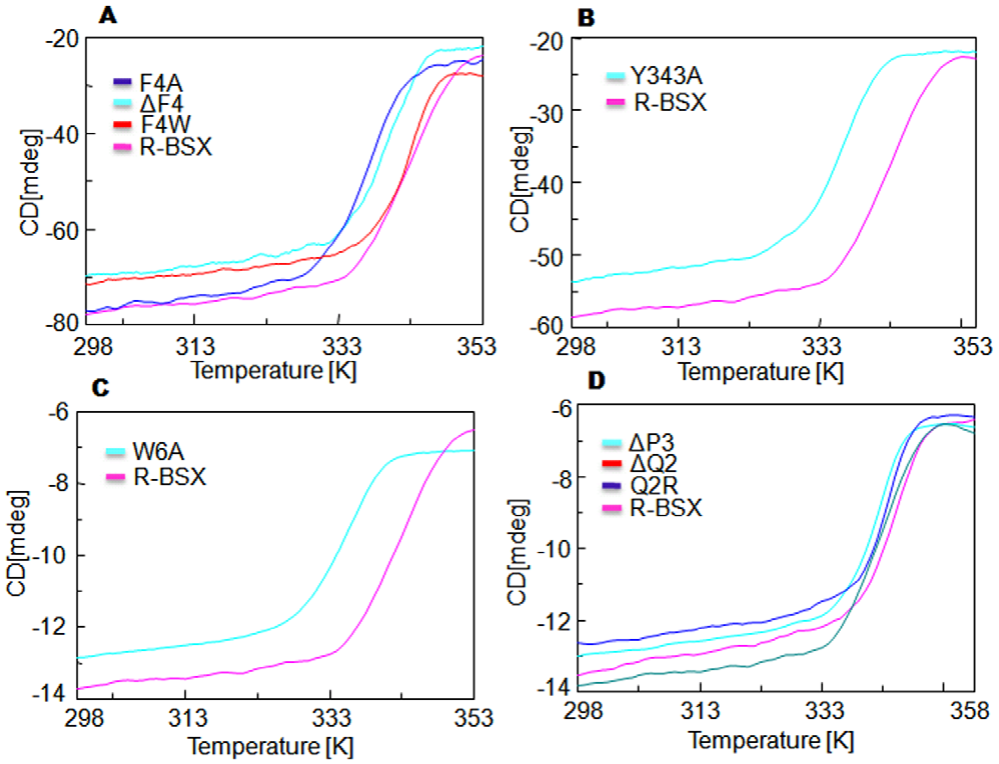
Thermal unfolding of R-BSX and its mutants using CD analysis. The thermal unfolding profile of deletion/substitution mutants of Phe4 (A), Y343 (B) and W6 (C) is shown. The thermal unfolding profile of Gln2 and Pro3 mutants is shown (D). The F4A, ΔF4, Y343A, W6A, and ΔW6 proteins unfolded faster than R-BSX. The ΔP3 mutant unfolded marginally faster, whereas the unfolding profiles of the Gln2 mutants remained similar to that of R-BSX.

To determine the role of the aromatic residues in protein stability at high temperatures, the thermal unfolding curve of R-BSX and its derivatives was determined (Figure 2). Native BSX (from *Bacillus sp.* NG-27) and R-BSX (expressed in *E.coli*) were found to unfold in an irreversible manner, and only the apparent T_m_ was calculated for all protein samples with a constant temperature slope of 333K/hr. The thermal unfolding curves for ΔF4 and F4A indicate the start of protein unfolding at 333K (Figure 2A), and the calculated ΔT_m_ for both mutants was ∼278K lower than that of R-BSX. Interestingly, the F4W mutant did not display any change in its thermal unfolding pattern, which was similar to that of R-BSX (Figure 2A). This native like stability of the F4W mutant can be explained because of the aromatic nature of the Trp residue. A T_m_ of ∼283K less was calculated from the thermal unfolding curve of the Y343A and W6A mutants (Figure 2B and 2C). The difference in ΔT_m_ was even more prominent for the ΔW6 and W6A mutants (Figure 2C). The W6A and Y343A mutants displayed similar thermal unfolding profiles, which could indicate that the stabilizing effect of the aromatic stacking interaction between Trp6 & Tyr343 was necessary. Deletion of Pro3 (ΔP3 mutant) marginally decreased the T_m_ by 274K, whereas the thermal unfolding curve of the ΔQ2 and Q2R mutants did not display any change (Figure 2D).

We observed a cloudy appearance of the protein samples at the end of the CD scan only for the ΔW6 and ΔY343 mutants. Differential scanning calorimetry (DSC) was carried out for selected mutants (ΔF4 and ΔW6) to look into possible aggregation of the protein, and R-BSX was used as a control. DSC analysis showed that aggregation was observed only for the ΔW6 mutant, as shown by the presence of exothermic C_p_ signals toward the end of the scan (Figure S2).

### Effect of temperature on protein activity

**Figure 3:**
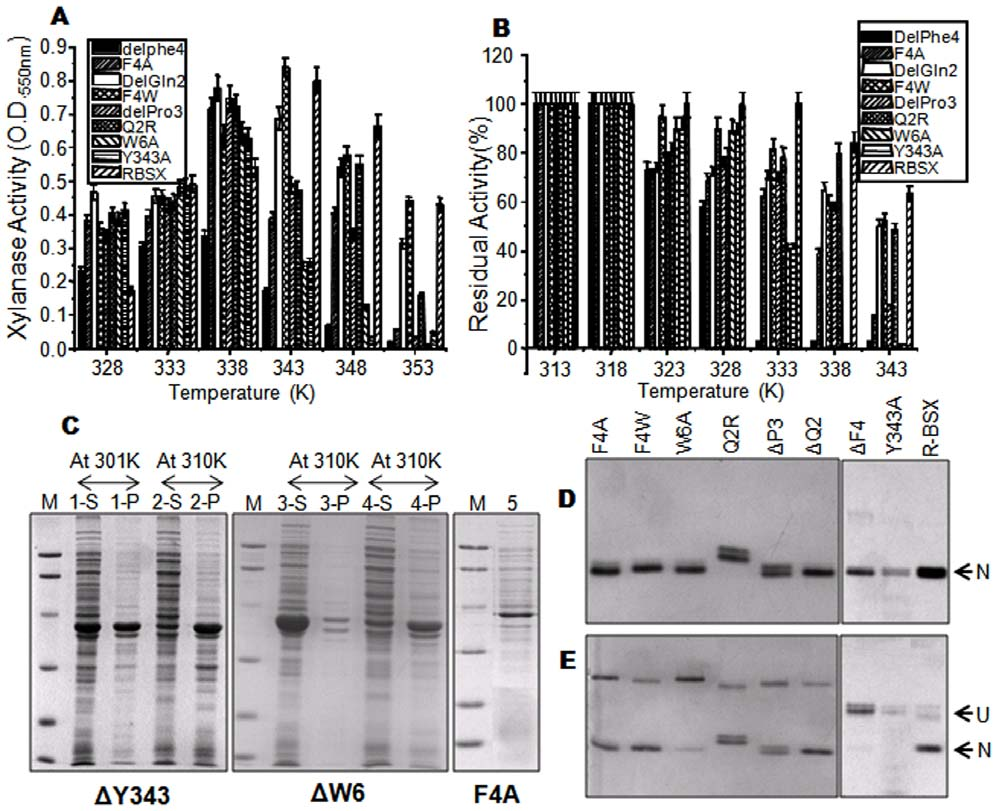
Activity, expression and native PAGE profiles of various mutants. (A) Xylanase activity profile of R-BSX mutants at various temperatures. All of the mutants except for F4W showed maximal activity at 338K. F4W showed optimal activity at 343K, similar to R-BSX. (B) Lanes 1 (S &P) and 2 (S&P) show the expression profile of ΔY343 at 301K and 310K, respectively. Lanes 3 (S &P) and 4 (S&P) show the expression profile of ΔW6 at 301K and 310K, respectively. Lane 5 shows the reduced expression level of F4A at 310K. “S” and “P” denote the soluble and pellet fraction after sonication. (C) Thermostability of R-BSX and its mutants. Samples were incubated for 15 min at various temperatures and assayed for residual xylanase activity at their respective optimal temperatures. (D) and (E) show native-PAGE profiles of thermal unfolding of protein samples at room temperature and at 343K, respectively. “N” & “U” denote the native and unfolded conformations, respectively. ΔF4, ΔW6 and W6A unfolded completely after incubation at 343K for 15 min. Note the slow migration of the Q2R mutant on native PAGE, which indicates some local conformational changes.

All derivatives of R-BSX displayed optimal activity at 338K, which is 278K lower than the optimal temperature for R-BSX (Figure 3A). The ΔF4 mutant displayed less enzyme activity than R-BSX. Although ΔQ2 and F4W have shown comparable activity at 338–343K, 338K was chosen to maintain homogeneity and experimental convenience (Figure 3A). To determine thermal stability, all of the mutants except ΔW6 and ΔY343 were incubated without substrate at various temperatures for 15 minutes, followed by measuring the residual activity at 338K (Figure 3B). ΔF4 was completely inactivated at 333K after 15 minutes of incubation. F4A retained more than 60% of its activity at a temperature up to 333K, but was inactive after a 15 minute incubation at 343K. Although the thermal unfolding curves of ΔF4 and F4A were quite similar, ΔF4 exhibited a lower amount of enzymatic activity. The native-like stability of F4W can be attributed to the fact that replacement of Phe4 with Trp does not disturb the aromatic interaction of this cluster. The W6A mutant displayed native-like xylanase activity and retained more than 80% residual activity at temperatures up to 328K, but lost all of its activity after a 15 minute incubation at 338K. The ΔP3 mutant showed almost 50% residual activity at temperatures up to 338K and became inactive at 343K. The ΔQ2 and Q2R mutants showed no significant change in their activity profile compared with R-BSX. All xylanase activity assay data correlated well with the thermal unfolding curves of the respective mutants, except for F4A. These data show that deletion/substitution of Gln2 does not affect R-BSX stability or activity at higher temperatures. On the other hand, substitution of Phe4, Trp6 or Tyr343 with alanine significantly decreased the stability and activity of R-BSX at higher temperatures. Deletion of Pro3 was also found to affect thermal stability, although to a lesser extent.

### Protein folding and accumulation

R-BSX and its mutants were expressed in their soluble forms when bacteria were grown at 310K, although the expression level of the F4A mutant was very low compared with that of R-BSX (Figure 3C, lane 5). Notably, ΔW6 and ΔY343 mutants localized mainly in inclusion bodies when the *E. coli* culture was grown at 310K (Figure 3C, lanes 2 & 4, respectively). They remained in their soluble forms when the growth temperature was reduced to 301K (Figure 3C, lanes 1 & 3, respectively), but they did not show any xylanase activity, which demonstrates that both ΔW6 and ΔY343 mutants have a mis-folded conformation.

### Native PAGE analysis of thermal unfolding

Measuring the difference in the mobility of native and heat denatured BSX and R-BSX using native PAGE analysis is a useful tool to distinguish the native and denatured protein structure [9]. Thus, native PAGE analysis was performed to visualize the thermal unfolding of R-BSX and its mutants at various temperatures. Protein samples were incubated at various temperatures for 15 minutes and then kept on ice before being subjected to native PAGE. Figures 3D and 3E show the native gel profiles of all samples incubated at room temperature (298K) and 343K, respectively. The Q2R mutant migrated as a doublet with less mobility than R-BSX, whereas the other mutants and R-BSX migrated with uniform mobility when samples were incubated at 298K (Figure 3D). The ΔF4, ΔW6, W6A and Y343A mutants unfolded almost completely at 343K (Figure 3E), resulting in the complete transition of the native form to the unfolded form. In contrast, the remaining mutants followed the same migration pattern as R-BSX at both temperatures. None of the heat denatured protein samples regained enzyme activity, indicating irreversible unfolding. At the same time, we did not observe any aggregate formation of protein samples when tested using DSC except for ΔW6 and ΔY343 mutants, which indicates that decreased mobility of heated protein samples could be due to the unfolding of protein molecules rather than protein aggregation (Figure S2). The results obtained from our native PAGE analysis of thermal unfolding were in good agreement with the CD thermal unfolding data.

### Tolerance to Proteinase K

**Figure 4:**
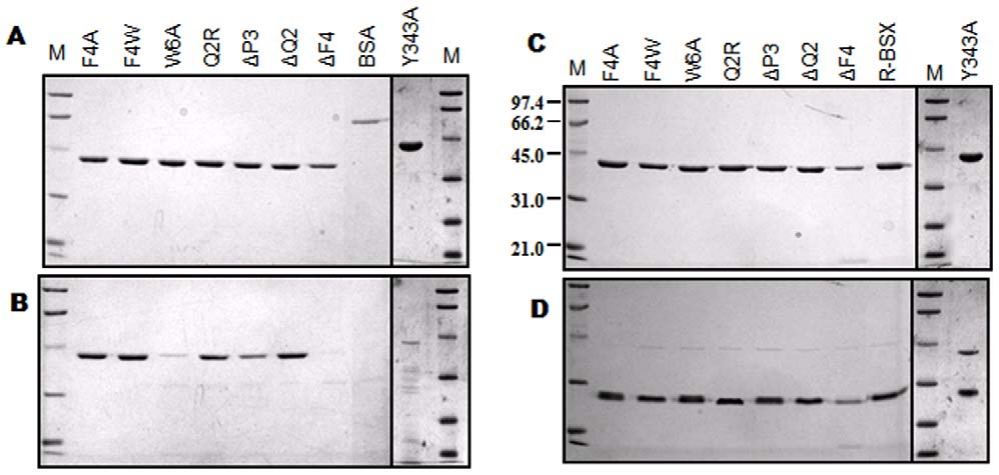
Effect of Proteinase K. The SDS-PAGE profiles of all proteins in the absence (A) and presence (B) of proteinase K. Samples were incubated with proteinase K for 24 hours at 301K. Note that all samples except W6A, ΔF4 and Y343A showed proteinase K resistance. The SDS-stability profiles of protein samples subjected to SDS-PAGE with (C) or without (D) prior boiling for 15 min are shown. All samples were incubated for 15 min in the presence of loading buffer containing SDS before they were subjected to SDS-PAGE.

To study the structural rigidity of R-BSX and its mutants, samples were subjected to proteinase K treatment at 301K for 24 hrs. Protease stability was determined by comparing the SDS-PAGE profiles of all samples before and after proteinase K treatment (Figure 4A & 4B). As shown in Figure 4B, the protein bands of the ΔF4, W6A and Y343A mutants disappeared completely after proteinase K treatment. GETAREA analysis (Table 2) shows that in the F-W-Y aromatic cluster, Phe4, Trp6 and Tyr343 are 34.9%, 15.5% and 4.5% surface exposed, respectively. Because Trp6 and Tyr343 are buried, they are expected to make a significant contribution to the conformational stability of R-BSX, which in turn affects protease stability. Proteinase K degradation of the W6A and Y343A mutants fell along the same lines. The deletion mutant of the partially exposed Phe4 (ΔF4) was proteinase K – sensitive, but the F4A and F4W mutants showed proteinase K tolerance similar to that of R-BSX. The ΔP3 mutant was also sensitive to proteinase K, but its rate of degradation was slow, as demonstrated by the presence of a faint protein band after digestion. The Gln2 deletion mutant (ΔQ2) and the SDS-sensitive Q2R mutant did not show any sensitivity to proteinase K, which again shows that Gln2 does not contribute to BSX stability and that its deletion/substitution did not dramatically change protein stability. The SDS-PAGE profiles of R-BSX mutants without Proteinase K treatment are shown in Figure 4A.

**Table 2:** GETAREA output for BSX N- and C-terminus residues.

Residue	Position	Total	Apolar	Backbone	Sidechain	Ratio(%)
Gln (2)	2	71.86	25.20	1.42	70.44	49.0
Pro (3)	3	48.98	48.98	8.00	40.98	39.0
Phe (4)	4	62.80	62.80	0.00	62.80	34.9
Trp (6)	6	47.05	26.99	12.25	34.80	15.5
Tyr (343)	343	8.74	2.12	0.00	8.74	4.50
Arg (344)	344	68.67	20.39	0.00	68.67	35.1

### SDS stability

The effect of SDS on the conformational stability of R-BSX mutants was assayed by comparing the migration of heated and unheated protein samples on SDS-PAGE (Figure 4C & 4D). As shown in Figure 4C, heated samples migrated corresponding to their molecular weight and the unheated samples migrated faster with higher mobility than the heated samples (Figure 4D). The higher mobility of the unheated samples can be explained on the basis of their monomeric compact structure, which permits faster migration than the heat denatured linear polypeptide chain. All of the samples showed SDS resistance and migrated faster on SDS-PAGE than the heat denatured samples under the tested conditions.

**Figure 5:**
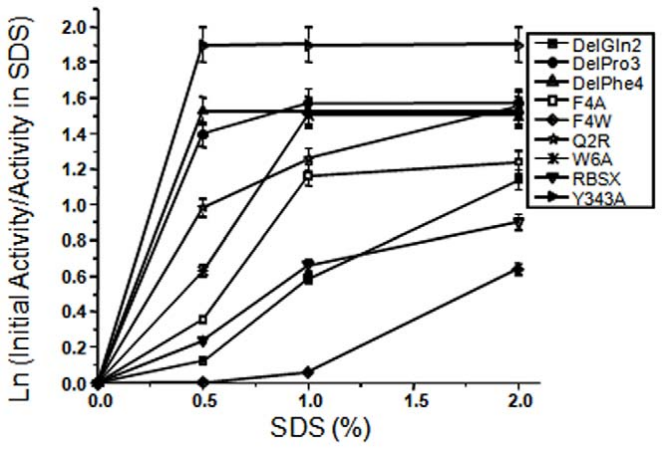
Xylanase activity in the presence of SDS. Samples were incubated for 12 h in the presence of various concentrations of SDS prior to submission for the activity assay. F4W displayed maximum stability in the presence of SDS, whereas the ΔQ2 mutant showed SDS stability comparable to that of R-BSX. The other mutants were more SDS sensitive and did not show any xylanase activity in the presence of ≥1% SDS.

To measure the effect of SDS on xylanase activity, we incubated the protein samples under various concentrations of SDS at room temperature for 12 hours, followed by the determination of xylanase activity (Figure 5). All of the protein samples, except for ΔQ2 and F4W, were more sensitive to SDS and completely inactivated in the presence of 1% SDS. Y343A, ΔP3, ΔF4 and W6A mutants showed maximal sensitivity to SDS, with no enzyme activity observed in the presence of 1% SDS. Surprisingly, Q2R was more sensitive to SDS than ΔQ2, which is equally SDS-stable as R-BSX. Interestingly, F4W displayed a higher SDS stability than that of R-BSX and retained more than 90% of its activity in the presence of 1% SDS after 12 hours of incubation. The F4W mutant was equally thermostable with enhanced SDS stability. This higher stability under poly-extreme conditions can be attributed to the better packing of the N- and C-terminal region. The SDS-PAGE and xylanase activity profiles showed that all the mutants are initially SDS-stable and retain their tertiary structure when incubated for a short time in the presence of SDS (Fig 4D), but they all respond differently when incubated for longer time periods (Figure 5).

### Pulse proteolysis

The amount of intact R-BSX and mutant proteins remaining after a 1 min proteolysis pulse with 0.15 mg/ml proteinase K was measured by SDS-PAGE (Figure S3). R-BSX seemed completely resistant to proteolysis up to a 4 M urea concentration, and they degraded slowly between 4 and 6 M urea. The fraction of remaining protein decreased rapidly in 6–8 M urea. The denaturation concentration at which half of the R-BSX is unfolded (C_m_) was determined to be 32.76±0.05M. F4W, which is the most stable mutant of R-BSX, showed complete resistance to proteolysis up to 3 M urea and degraded rapidly in 4–6 M urea. The C_m_ value for F4W was determined to be 5.0±0.05 M. At concentrations of urea below 2 M, F4A and Y343A seemed resistant to proteolysis. The C_m_ value for F4A and Y343A was calculated to be 4.25 and 3.0±0.05 M, respectively. The C_m_ value for ΔQ2, Q2R, and ΔP3 mutants was calculated to be 21.85, 19.84 and 17.94±0.05 M, respectively. The W6A and ΔF4 mutants showed substantial proteolysis of folded protein with a 1 min pulse even with no urea; therefore, a pulse proteolysis experiment was not performed for these mutants. The global stability of the protein, ΔG_unf_^o^, in water was calculated by multiplying the C_m_ by the *m*-values as described previously (Table 3).

**Table 3:** Determination of ΔG_unf_^o^ for R-BSX and its mutants using pulse proteolysis.

Sample	C_m_	ΔG_unf_^o^
R-BSX	7.1	32.7665
F4W	5.0	23.075
F4A	4.25	19.61
Y343A	3.0	13.845
ΔQ2	4.75	21.85
ΔP3	3.9	17.94
Q2R	4.3	19.84

### Limited proteolysis with trypsin

**Figure 6:**
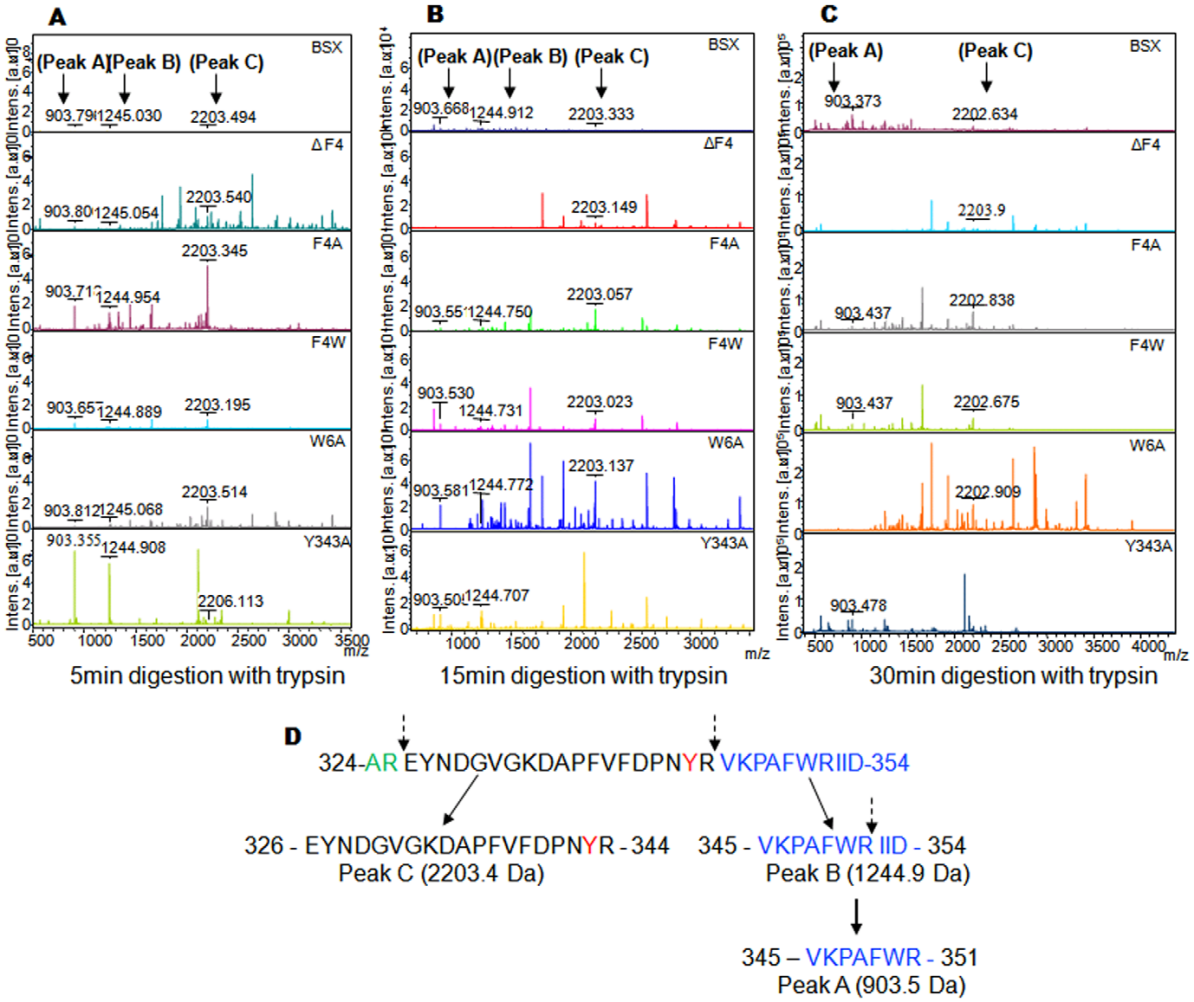
Limited proteolysis profile of BSX and its mutant. All protein samples were incubated with trypsin for 5 min (A), 10 min (B) and 15 min (C) under native conditions and analyzed by mass spectrometry. (D) Schematic representation of limited proteolysis of R-BSX by trypsin toward the C-terminus. A partial C-terminal sequence of R-BSX from 326 to 354 is shown. Tyrosine 343 involved in the aromatic stacking interaction is shown in red. The dashed arrow indicates the trypsin cleavage sites.

Detailed investigations on the structural features of native BSX and its mutants were carried out using limited proteolysis followed by mass spectrometry analysis. Native BSX was found to exhibit considerable resistance against proteolytic digestion with trypsin under native conditions. Hence, trypsin was used as a probe to detect any structural changes due to the deletion or substitution of the respective amino acid. BSX and its mutants were subjected to limited trypsin proteolysis for various time periods and tryptic peptide mass maps were analyzed. As shown in figure 6A–C, comparative analyses of peptide mass fingerprints revealed that the intensity of peak-A (903.5Da) increased with time for stable mutants including BSX, whereas the intensity of peak-A decreased with time for sensitive mutants. Sequence analysis of this peak revealed that this fragment contained ^345^VKPAFWR^351^, which corresponds to the C-terminal region of the protein. Another highlighted peak named as peak-B (1244.9Da) was found, which corresponds to the ^345^VKPAFWRIID^354^ region. As peak-A and peak-B share the same cleavage point (Arg-344), peak-A is the most probable product of peak-B after tryptic cleavage at Arg-351. Another highlighted peak, peak-C (2203.4Da), originated from the fragment ^326^EYNDGVGKDAPFVFDPNYR^344^, also from the C-terminal region of the protein. The intensity of Peak-C also followed a similar pattern to that of peak-A for stable and sensitive mutants.

## Discussion

Many factors affect protein stability, including hydrophobic clusters [2], oligomerization [25], [ 26], salt bridges [25], [ 27], [ 28], an increased number of side-chain–side-chain hydrogen bonds, a smaller number of glutamine and asparagines, improved packing of the hydrophobic core [29], aromatic clusters [4], [ 30], [ 31], [ 32], and cation-pi interactions [31], [ 33], [ 34]. A comprehensive *in silico* analysis by Gromiha et al. showed that most of the bona fide stabilizing residues are oriented toward the interior of the TIM-barrel fold [35] but did not predict much about the interactions between the very N- and C-terminal residues in the stability of these proteins. In this study, for the first time we have experimentally established the crucial role played by the N- and C-terminal contacts involving Phe4-Trp6-Tyr343 (F-W-Y) aromatic cluster in folding, as well as the stability of BSX under extreme conditions.

As per the BSX crystal structure, the F4-W6 and W6-Y343 aromatic pairs are arranged respectively in an offset and edge-face geometry. Additionally, F4 and W6 were found to be involved in the N- to C-terminus (N-C) interaction via the W6-Y343 aromatic and the F4-R344 cation-pi interactions. In BSX, when the F-W-Y cluster was disrupted with deletions/Ala-substitutions (of Phe4, Trp6, and Tyr343), there were profound effects on the stability of these mutants under different stress conditions.

**Figure 7:**
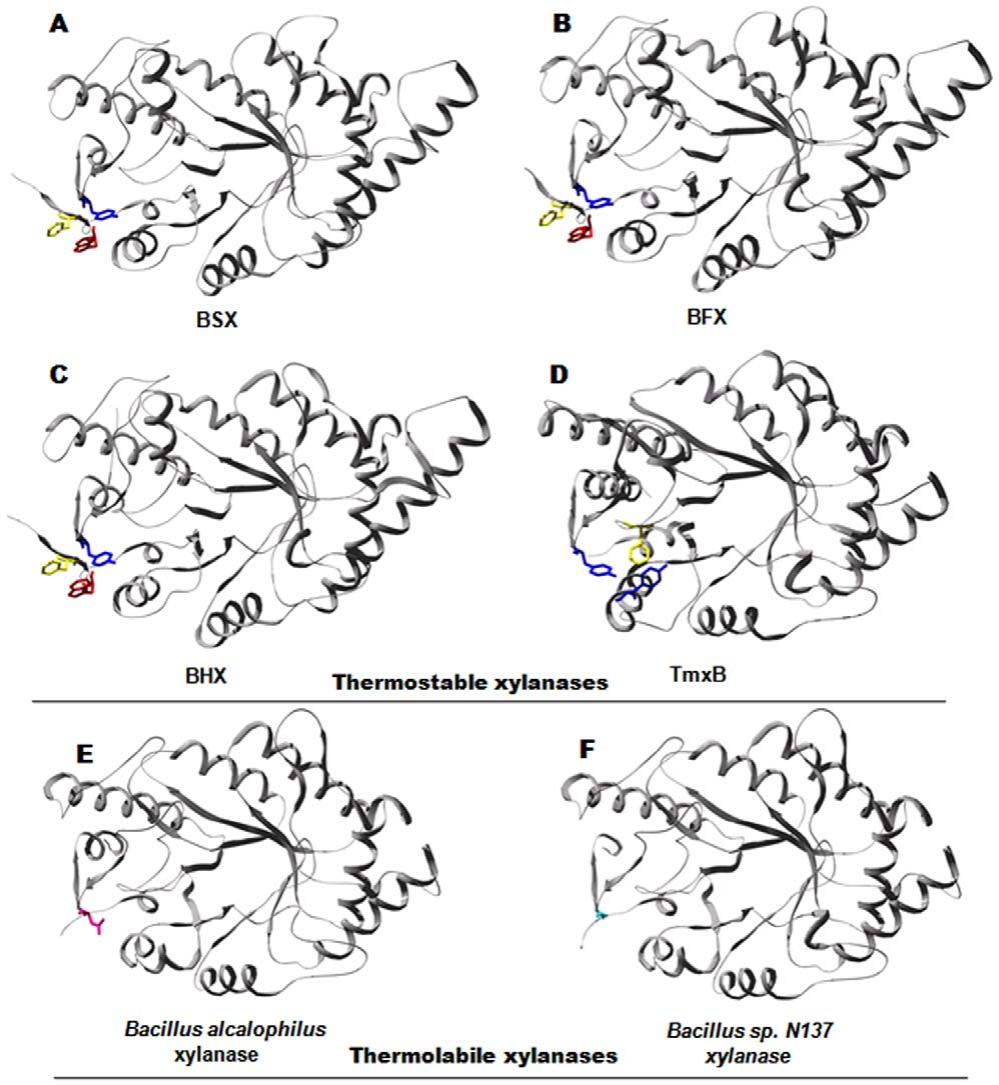
Interaction between N- and C-terminal regions. Structures of BFX (B), BHX (C) and TmxB (D) showed the presence of aromatic clusters equivalent to the studied F-W-Y cluster of BSX (A), which is reported as thermostable in the literature. E and F clearly show that xylanase from *Bacillus alcalophilus* and *Bacillus sp. N137* does not contain any aromatic cluster to hold its termini together, and this might be the reason for its thermolabile nature. Tyr343 (Shown in blue) of BSX was found to be conserved in BFX, BHX and TmxB. Xylanases from *Bacillus alcalophilus* and *Bacillus sp. N137* were found to contain Asn (magenta) and Gly (Cyn) as topologically equivalent residues, respectively. Phe and Trp residues are shown in yellow and red, respectively.

Structural alignment of BSX with BHX [22] and BFX [23] revealed the presence of similar aromatic clusters at their N- and C-terminus, and this might be the reason behind their similar optimal temperatures (Figure 7A–D). Structural analysis of hyper-thermostable TmxB [36] also indicated that Tyr820 (equivalent to Tyr343 of BSX) is involved in aromatic interaction with Tyr547 and Phe796, making N- and C-terminal interactions (Figure 7D). On the other hand, an aromatic cluster equivalent to the F-W-Y cluster of BSX was absent in the xylanases from *Bacillus* N137 and *alcalophilus*, which are thermolabile in nature (Figure 7E–F). Our results show that these aromatic cluster interactions involving N- to C-terminal residues are a common feature of GH10 thermostable xylanases from *Bacillus sp*.

Of all the mutants, the deletion mutants of Trp6 (ΔW6) and Tyr343 (ΔY343) were affected the most in terms of enzyme activity, stability and folding. Deletion of Phe4 (ΔF4) did not affect protein folding and, as a result, the ΔF4 mutant showed xylanase activity. The fact that Phe4 is surface exposed (34.9%, Table 2) could account for this. The removal of Phe4 does not affect protein folding as severely as the removal of either W6 or Y343, both of which are buried inside the protein (Table 2). Additionally, the deletion mutants of BSX (ΔW6, ΔF4 & ΔY343) cause the repositioning of all the preceding residues with respect to the rest of the protein, which could be either destabilizing or stabilizing in nature and hence may be responsible for the drastic loss of stability and folding of these deletion mutants. Proteins have a dominant tendency to bring their N- and C-terminal elements together and the docking of their terminal element is the first step in folding [37], [ 38], [ 39]. In light of this, the non-native conformation of ΔW6 and ΔY343 mutants is suggestive of the fact that besides providing structural stability, interactions between the very N- and C-terminal residues via the F-W-Y aromatic cluster are perhaps important for the initial events in BSX folding. The W6A and Y343A mutants were found to be sensitive under all the conditions tested, indicating that disrupting the aromatic interactions of the F-W-Y cluster promoted the local unfolding in this region, verifying the stabilizing nature of these residues in the native enzyme. Generally, surface residues of a protein are widely regarded to be tolerant to substitution because exposed sites remain exposed in both native and denatured states. However, several studies have shown that the substitution of an amino acid(s) on the protein surface has different effects on its stability depending on the environment of the mutation site(s) [40], [ 41], [ 42]. Our engineering work on BSX demonstrates that single point mutants of the partially exposed Phe4 (34.9%, Table 2) are sufficient to drastically modulate the stability of this enzyme under poly-extreme conditions, as both ΔF4 and F4A mutants showed decreased thermal and SDS stability compared with R-BSX. However, only ΔF4 was found to be proteinase K sensitive. Given that Phe4 is involved in two major long-range interactions including aromatic stacking with Trp6 and cation-pi interaction with Arg344, it is not surprising that the deletion/Ala-substitution mutants of Phe4 were less stable than the wild type under poly-extreme conditions because of the loss of both of these interactions. It may be noted here that in a previous study on the cold shock protein B from *Bacillus subtilis*, replacement of a partially exposed phenylalanine by alanine was shown to destabilize the folded state [43]. We also observed a significant decrease in the in vivo expression profile for the F4A mutant. Because the F4A mutant was found to have considerable tolerance against proteinase K, the possibility that this is due to protein degradation is not likely. Another study on α-amylase (BLA) from *Bacillus licheniformis* has shown that mutating a surface-exposed Asn190 residue with Phenylalanine stabilized the BLA by creating triple aromatic interactions [44]. In contrast to F4A, the F4W mutant showed native-like thermal stability, proteinase K stability and a much higher tolerance to SDS denaturation. This could be the result of better packing of its N- and C-terminal region due to the aromatic nature of the Trp6 residue. It further strengthens the argument that surface-exposed residues (such as Phe4) are critical for protein stability. In addition, the reduced thermal, SDS, and Proteinase K stability of ΔP3 mutant could be attributed to its lower conformational entropy [45].

Limited proteolysis has been used previously to investigate structure-function relationships in proteins [46], [ 47]. Proteolysis of a protein can occur only if the polypeptide chain can bind and adapt the specific stereochemistry of the protease active site [48]. Therefore, the proteolysis of the folded proteins is much slower, unless the folded proteins have intrinsically unstructured regions [18]. BSX has shown high levels of tolerance against proteolytic digestion by trypsin under native conditions, which is the most commonly used enzyme in mass spectrometry for protein digestion with high substrate specificity. Structural analysis of BSX has shown that Arg344 is present in the close vicinity of the studied aromatic cluster (Fig 1A), allowing us to use trypsin to probe protease-accessibility of Arg344 due to the deletion/substitution of the respective residue in the intact protein molecules. Mass spectrometry analyses of time-dependent tryptic digested peptides of BSX and its mutants clearly showed that ΔF4, F4A, W6A and Y343A mutant proteins were more sensitive to trypsin proteolytic digest compared to BSX. Although Arg344 was found to be 35.1% surface exposed in BSX, the residue is still quite inaccessible to trypsin, which makes BSX tolerant to proteolytic digestion by trypsin (Table 2). As Arg344 is involved in cation-pi interaction with Phe4, the appearance of peak-A after 5 min of incubation clearly suggests that Arg344 is much more exposed and susceptible to proteolytic digestion by trypsin in ΔF4 and F4A mutants compared to BSX and the F4W mutant, which are tolerant to trypsin proteolytic digestion. GETAREA analysis of ΔF4 and F4A mutant proteins showed that Arg 344 is 54.3% and 49.6% exposed in these mutants, respectively, compared to BSX, where Arg 344 is only exposed 35.1% (Table S1). In the case of the W6A mutant, Phe4 was found to be 14.2% more surface exposed, which might be affecting its interaction with Arg344 and probably explains its sensitivity to trypsin proteolytic digestion. The GETAREA results further supported the limited proteolysis data, suggesting that trypsin proteolytic digestion of Arg344 is directly proportional to its surface-exposed nature. Limited proteolysis experiments also enabled us to identify the presence of expected peak A, which emerged very early (within 5min) during the tryptic digestion of the susceptible mutants compared to the stable mutants, where it appeared after only 15 min of digestion. A close examination of this peptide indicated that peak-A can be digested further by trypsin at Lys-346 followed by Pro347 to generate a peptide of 672 Da having sequence of ^346^PAFWR^351^ [49]. However, we could not find the exact reason for the decreasing intensity of peak-A with increasing tryptic digestion time. The fast degradation rate of peak-A clearly explains why this peak cannot be seen during the complete proteolysis. In the light of these results, it seems that tryptic cleavage of Arg344 is one of the initial events during trypsin digestion of R-BSX, and eventually the R-BSX was digested further with time (Figure 6D). Limited proteolystic analysis provided an interesting clue about the proteolysis mechanism of these mutants.

Another important observation was that the deletion/substitution mutants of non-aromatic residues such as Gln2 and Pro3 did not show any comprehensive change in stability under all of the conditions tested. The reduced SDS stability of Q2R could be because SDS is known to interact with cationic residues [50], such as Arg and Lys and hence replacing 49% of the surface-exposed Gln2 residue with Arg could actually facilitate the process of unfolding the Q2R mutant protein in the presence of SDS. The reduced mobility of Q2R in native PAGE analysis could also be due to the local unfolding caused by the introduction of Arg at this position. Our results clearly demonstrate the role of aromatic interactions in protein stability under poly-extreme conditions, as well as in facilitating earlier events required for BSX folding, which are achieved by the ability of these aromatic interactions to form N- to C- terminal contacts. These results also suggest that stability for one set of extreme conditions can help the molecule adapt to another set of extreme conditions. The deletion/substitution of Phe4, Trp6 and Tyr343 dramatically affected the stability of BSX under poly-extreme conditions, whereas the deletion/substitution of Gln2 and Pro3, which are not part of the F-W-Y cluster, had only a mild effect on the stability of BSX (Fig 2D). However, the contribution of surface-exposed Gln2 and Pro3 cannot be completely ruled out. In conclusion, our study has revealed, for the first time, the role of the interaction between the very N- and C- terminal residues in protein stability of the TIM-barrel fold of GH10 xylanases, and this has implications for improving the stability of other TIM-barrel fold proteins under sub-optimal conditions.

## Supporting Information

Figure S1 Multiple sequence alignments of the BSX with those of other GH10 xylanases of Bacillus origin. AAB70918: Bacillus sp. NG-27 (BSX), which is used in the present study, AAV98623: Bacillus halodurans S7 (BHX), AAQ83581: Bacillus firmus (BFX), CAA84631: Bacillus sp. N137, AAQ99279: Bacillus alcalophilus AX2000. Aromatic residues enclosed in rectangles are involved in F-W-Y aromatic cluster formation and are present only in the thermostable BSX, BHX and BFX proteins.
(0.58 MB DOC)

Figure S2 DSC profile of R-BSX, _F4 and _W6. Figure 3A–C shows the DSC thermogram of R-BSX, _F4 and _W6. We used 0.1mg/ml of protein sample in 10mM Phosphate buffer, pH 7.0 with a heating rate of 333K/hr. in figure 3C, we can clearly see the aggregation during thermal unfolding of _W6 mutant which is indicated by the exothermic flow at the end of the denaturation process. Figure 3A′–C′ shows the reversibility of all three protein samples under same experimental conditions mentioned above. All the thermodynamic parameters observed by DSC are indicated in panels along with the respective curve.
(1.04 MB DOC)

Figure S3 Determination of Cm of R-BSX and its mutant by pulse proteolysis. We used 0.15mg/ml of proteinase K to digest 0.3mg/ml R-BSX and its mutants equilibrated in 20mM Tris-Cl,(pH 8.5) and urea (0–8M). Figure 2A–G shows the SDS-PAGE gel profile and ffold of R-BSX and its mutant after 1min pulse proteolysis. (H) _F4 mutant showed substantial proteolysis at 0M urea concentration (Lane -3). (I) W6A mutant also showed substantial proteolysis at 0M urea concentration. In panel H and I, “U” denotes the protein sample without protease treatment.
(3.15 MB DOC)

Table S1 GETAREA output for N- and C-terminus residues of BSX mutants. Mutated residues are shown in bold letter. Significant change in surface accessibility of any residue is shown in red color.
(0.06 MB DOC)
