# Supplementary figures and images for: The Critical Role of N- and C-Terminal Contact in Protein Stability and Folding of a Family 10 Xylanase under Extreme Conditions
Source: PLoS One. 2010 Jun 28;5(6):e11347. doi: 10.1371/journal.pone.0011347 (PMC2893209; doi:10.1371/journal.pone.0011347)

**Figure S1**


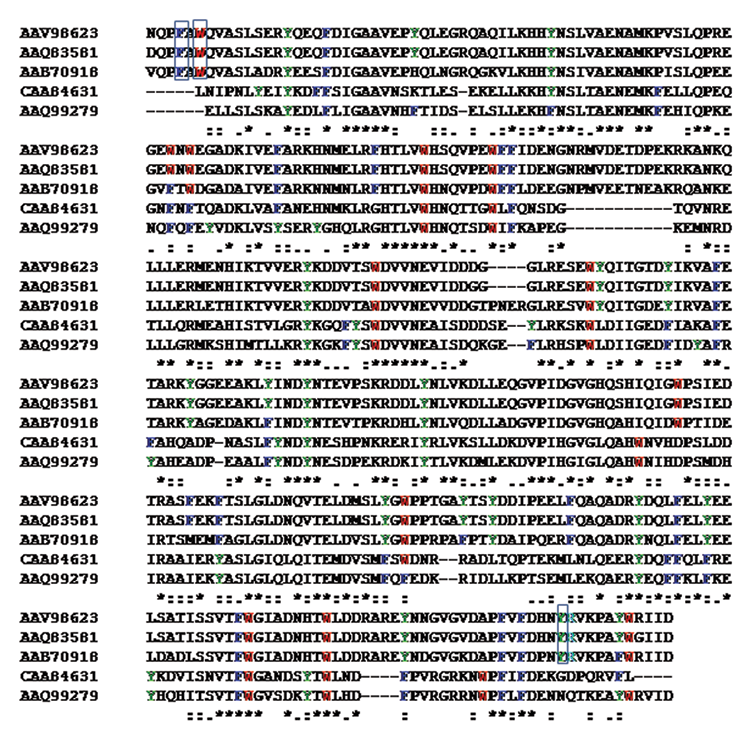

Supplement: Figure S1 — Multiple sequence alignments of the BSX with those of other GH10 xylanases of Bacillus origin. AAB70918: Bacillus sp. NG-27 (BSX), which is used in the present study, AAV98623: Bacillus halodurans S7 (BHX), AAQ83581: Bacillus firmus (BFX), CAA84631: Bacillus sp. N137, AAQ99279: Bacillus alcalophilus AX2000. Aromatic residues enclosed in rectangles are involved in F-W-Y aromatic cluster formation and are present only in the thermostable BSX, BHX and BFX proteins. (0.58 MB DOC) [file pone.0011347.s001.doc]
