# Supplementary material for: The Critical Role of N- and C-Terminal Contact in Protein Stability and Folding of a Family 10 Xylanase under Extreme Conditions
Source: PLoS One. 2010 Jun 28;5(6):e11347. doi: 10.1371/journal.pone.0011347 (PMC2893209; doi:10.1371/journal.pone.0011347)
Supplement: Table S1 — GETAREA output for N- and C-terminus residues of BSX mutants. Mutated residues are shown in bold letter. Significant change in surface accessibility of any residue is shown in red color. (0.06 MB DOC) [file pone.0011347.s004.doc]

**Table S1**

| **ΔF4 Mutant** | | | | | | |
| --- | --- | --- | --- | --- | --- | --- |
| **Residue** | **Position** | **Total** | **Apolar** | **Backbone** | **Sidechain** | **Ratio(%)** |
| Gln | 2 | 71.86 | 25.20 | 1.42 | 70.44 | 49.0 |
| Pro | 3 | 52.41 | 52.41 | 11.43 | 40.98 | 39.0 |
| **Phe** | 4 | Deleted | | | | |
| Trp | 6 | 77.56 | 54.13 | 12.25 | 65.31 | 29.1 |
| Tyr | 343 | 10.56 | 2.24 | 1.71 | 8.85 | 4.6 |
| Arg | 344 | 106.21 | 33.17 | 0.01 | 106.20 | 54.3 |
| **F4A Mutant** | | | | | | |
| Gln | 2 | 71.86 | 25.20 | 1.42 | 70.44 | 49.0 |
| Pro | 3 | 49.00 | 49.00 | 8.01 | 40.98 | 39.0 |
| **Ala** | 4 | 10.16 | 10.16 | 0.00 | 10.16 | 15.6 |
| Trp | 6 | 63.25 | 40.93 | 12.25 | 51.00 | 22.7 |
| Tyr | 343 | 8.74 | 2.12 | 0.00 | 8.74 | 4.50 |
| Arg | 344 | 97.01 | 27.80 | 0.00 | 97.01 | 49.6 |
| **W6A Mutant** | | | | | | |
| Gln | 2 | 71.86 | 25.20 | 1.42 | 70.44 | 49.0 |
| Pro | 3 | 48.98 | 48.98 | 8.00 | 40.98 | 39.0 |
| Phe | 4 | 88.36 | 88.36 | 0.00 | 88.36 | 49.1 |
| **Ala** | 6 | 23.87 | 10.53 | 13.34 | 10.53 | 16.2 |
| Tyr | 343 | 12.33 | 5.71 | 0.00 | 12.33 | 6.4 |
| Arg | 344 | 68.67 | 20.39 | 0.00 | 68.67 | 35.1 |
| **Y343A Mutant** | | | | | | |
| Gln | 2 | 71.86 | 25.20 | 1.42 | 70.44 | 49.0 |
| Pro | 3 | 48.98 | 48.98 | 8.00 | 40.98 | 39.0 |
| Phe | 4 | 62.80 | 62.80 | 0.00 | 62.80 | 34.9 |
| Trp | 6 | 47.05 | 26.99 | 12.25 | 34.80 | 15.5 |
| **Ala** | 343 | 7.84 | 7.84 | 0.00 | 7.84 | 12.1 |
| Arg | 344 | 68.67 | 20.39 | 0.00 | 68.67 | 35.1 |
